# Independent Relationship of Changes in Death Rates with Changes in US Presidential Voting

**DOI:** 10.1007/s11606-018-4568-6

**Published:** 2018-09-05

**Authors:** Lee Goldman, Maribel P. Lim, Qixuan Chen, Peng Jin, Peter Muennig, Andrew Vagelos

**Affiliations:** 1 Vagelos College of Physicians and Surgeons, Columbia University, New York, NY USA; 2 Mailman School of Public Health, Columbia University, New York, NY USA

**Keywords:** age-adjusted death rate, presidential election, deaths of despair, rural public health, 2016 election

## Abstract

**Background:**

The outcome of the 2016 presidential election is commonly attributed to socioeconomic and ethnic/racial issues, but health issues, including “deaths of despair,” may also have contributed.

**Objective:**

To assess whether changes in age-adjusted death rates were independently associated with changes in presidential election voting in 2016 vs. 2008.

**Design:**

We used publicly available data in each of 3112 US counties to correlate changes in a county’s presidential voting in 2016 compared with 2008 with recent changes in its age-adjusted death rate, after controlling for population and rural-urban status, median age, race/ethnicity, income, education, unemployment rate, and health insurance rate.

**Design Setting:**

Cross-sectional analysis of county-specific data.

**Setting/Participants:**

All 3112 US counties.

**Main Measures:**

The independent correlation of a county’s change in age-adjusted death rate between 2000 and 2015 with its net percentage Republican gain or loss in the presidential election of 2016 vs. 2008.

**Key Results:**

In 2016, President Trump increased the Republican presidential vote percentage in 83.8% of counties compared with Senator McCain in 2008. Counties with an increased Republican vote percentage in 2016 vs. 2008 had a 15% higher 2015 age-adjusted death rate than counties with an increased Democratic vote percentage. Since 2000, overall death rates declined by less than half as much, and death rates from drugs, alcohol, and suicide increased 2.5 times as much in counties with Republican gains compared with counties with Democratic gains. In multivariable analyses, Republican net presidential gain in 2016 vs. 2008 was independently correlated with slower reductions in a county’s age-adjusted death rate. Although correlation cannot infer causality, modest reductions in death rates might theoretically have shifted Pennsylvania, Michigan, and Wisconsin to Secretary Clinton.

**Conclusions:**

Less of a reduction in age-adjusted death rates was an independent correlate of an increased Republican percentage vote in 2016 vs. 2008. Death rates may be markers of dissatisfactions and fears that influenced the 2016 Presidential election outcomes.

**Electronic supplementary material:**

The online version of this article (10.1007/s11606-018-4568-6) contains supplementary material, which is available to authorized users.

## INTRODUCTION

In 2016, President Trump achieved a surprising victory by winning the US electoral college despite losing the popular vote.^1^ Many factors may influence voting behavior, but a common argument is that he won by receiving more votes from people who have been left behind economically (especially older, less educated, more rural, white voters) and by emphasizing racial and ethnic controversies.^2^ Although the percentage of swing voters has reportedly diminished from about 15% to about 5%,^3^ it is these swing voters as well as voter turnout^4^ that drove election results in 2016.

Longevity has improved more slowly or actually worsened in many of the same demographic groups—Americans who are white, less educated, have lower incomes, and live in rural areas—that voted disproportionally for President Trump.^5–10^ Furthermore, these same Americans increasingly are victims of “deaths of despair” related to alcohol, drugs, and suicide.^10–12^ To assess the comparative importance of recent health changes vs. recent changes in demographic and socioeconomic factors on the 2016 presidential election, our goal was to determine whether there was an independent correlation of county-specific changes in death rates^13, 14^ with changes in voting, after adjusting for these other possible correlates.

## METHODS

### Data Sources

US county general election results were obtained from Dave Leip’s Atlas of US Presidential Elections.^1^ County-level population size, age, race, ethnicity, and mortality data were obtained from the Centers for Disease Control and Prevention (CDC) Wide-ranging Online Data for Epidemiologic Research (WONDER) online databases.^15, 16^ The US Census Bureau provided county-level data sets on urban-rural classification,^17^ income,^18^ unemployment,^19^ and educational attainment.^20^ Health insurance coverage estimates were graciously provided by Dr. Haomiao Jia and based on data obtained from the US Census Bureau.^21^

### Voting Patterns

Our analysis used county-level data of voting patterns from 3112 US counties (online Supplemental eTable 1).^22^ We first assessed the results of the 2016 presidential election by county. Next, we created a continuous outcome defined as the “net Republican percentage gain,” which was the sum of any improvement in the percentage of votes for President Trump in 2016 compared with Senator McCain in 2008 plus any decrement in the percentage of votes for Secretary Clinton in 2016 compared with President Obama in 2008 (for example, 229 counties had a positive net Republican gain even though the Republican presidential voting percentage decreased, because the Democratic percentage decreased by more).

### Potential Predictors of Voting Patterns

As potential predictors of voting change, we considered county-level demographic, socioeconomic, and health characteristics, including urban-rural status; population size; median age; the percentage of the population that was black/African-American, Hispanic/Latino, or Asian; median income; unemployment rate; educational level (defined as the percentage of the population age 25 years and older with a bachelor’s degree or higher); coverage by health insurance; and age-adjusted death rates. Although many counties are too small for a reliable county-level analysis of cause-specific death rates, we also summed the aggregate rates of “deaths of despair” related to alcohol, drugs, or suicide. We imputed age-adjusted rates of “deaths of despair” for counties with suppressed death counts by building a multilevel, left-censored, Bayesian imputation model (see online Supplemental Methods).^23^

### Statistical Analysis

Using random intercept models that accounted for clustering of counties within states, we compared means of these potential predictors among (a) counties won by President Trump vs. those won by Secretary Clinton in 2016 and (b) counties in which the percentage Republican vote for President Trump in 2016 was greater than for Senator McCain in 2008 vs. counties in which the percentage Democratic vote for Secretary Clinton in 2016 was greater than for President Obama in 2008. To examine whether the comparison between counties won by the Republican or Democratic candidates differed between the two elections, we concatenated the 2008 and 2016 election data and fit a 3-level random intercept model (elections/counties/states) that included election year and its interaction with the comparison group as covariates.

To assess the independent associations between voting patterns and potential predictors of voting change, we fitted a multivariable random effects model with the net Republican percentage gain as the outcome variable and a county’s urban/rural status, population size, and changes in age, race, income, unemployment, education, health insurance, and age-adjusted death rate from 2000 to 2015 as covariates. The model allowed state-specific intercept and state-specific slope associated with the age-adjusted death rates, where the random intercept and random slope were assumed to follow a bivariate normal distribution with non-zero covariance. A log10 transformation was applied to the county population size, and a piecewise model with the cutoff value at the population of 50,000 was used to model different population effects between counties with a population smaller or greater than 50,000. All potential predictors and their changes were analyzed as continuous variables except the urban/rural status. In a secondary analysis, we replicated the multivariable model but replaced the age-adjusted overall death rates with the multiply-imputed, age-adjusted rates of “deaths of despair” and of all other deaths. We conducted three sensitivity analyses: (1) multivariable model weighted by population size; (2) principal component analysis on the changes in age, race, income, unemployment, education, and health insurance from 2000 to 2015, with a multivariable model against age-adjusted death rates, urban/rural, population size, and principal components; (3) multivariable model with the net Republican gain between 2000 to 2016 elections as the outcome variable. All analyses were conducted in R version 3.3.1.^24^

## RESULTS

In 2016, Secretary Clinton received 65.9 million votes (48.0%) compared with 63 million votes (45.9%) for President Trump (Table 1). Since President Obama received 69.5 million votes in 2008, Secretary Clinton trailed him by 3.6 million (4.8%) votes. By comparison, President Trump outperformed Senator John McCain by 3.0 million votes but had only 0.3% more of the popular vote because other party candidates increased their percentage vote.

**Table 1:** Presidential Vote 2016 ^1, 15, 16, 18–21^

	Republican President Trump	Democratic Secretary Clinton	
Popular vote, millions (%)*	63.0 (45.9%)	65.9 (48.0%)	
Change in vote 2016 vs. 2008 (millions)	+ 3.0	− 3.6	
Change in % vote 2016 vs. 2008	+ 0.3%	− 4.8%	
Counties won	2622	490	
Other characteristics of counties won†	Mean (SD)	Mean (SD)	*p* value^‡^
Median age (years)	41.2 (5.0)	37.6 (5.4)	< 0.001
% white, not Hispanic or Latino	82.2 (15.3)	55.5 (24.9)	< 0.001
% black/African-American population	7.6 (10.5)	23.0 (23.9)	< 0.001
% Hispanic/Latino population	7.9 (11.1)	15.5 (21.1)	< 0.001
% Asian/Pacific Islander population	1.1 (1.2)	4.6 (7.3)	< 0.001
Median household income (in $1000s)	47.8 (10.7)	52.0 (18.3)	< 0.001
% unemployment	5.4 (1.8)	6.2 (2.4)	< 0.001
% of population over age 25 with bachelor’s degree or higher	19.2 (7.1)	29.7 (13.5)	< 0.001
% with health insurance	90.9 (3.7)	91.5 (3.8)	0.771
Age-adjusted death rate (per 100,000)	838.8 (148.2)	781.2 (190.1)	< 0.001
Age-adjusted rate of “deaths of despair” (per 100,000)^§^	45.6 (31.3)	40.5 (56.7)	< 0.001

*SD*, standard deviation

*8.3 million votes (6.0%) went to other candidates

†Data are from 2015 and exclude 88 counties with missing data on one or more of the county characteristics

‡*p* value based on *t* test in random intercept models that accounted for clustering of counties within states

^§^Using the multiply-imputed data on age-adjusted rate of “deaths of despair,” defined as ICD codes listed in online Supplemental eTable 7. The analysis accounts for imputation uncertainty using Rubin’s multiple imputation combining rules (see online Supplemental Methods)

President Trump exceeded the percentage votes for Senator McCain in 2607 (83.8%) of the 3112 US counties for which voting data were available, whereas Secretary Clinton exceeded President Obama’s percentage vote in only 108 counties (3.5%). Both 2016 candidates fell short of the votes received by their party’s 2008 candidate in 398 (12.8%) counties because of increased votes for third-party candidates, and both 2016 candidates increased their party’s percentage vote in one county. The median net Republican per-county gain was 15%, with a range of − 23 to + 69% (online Supplemental eFig. 1).

### Demographic and Socioeconomic Correlates of 2016 Presidential Voting

Counties won by President Trump were older; more white; had a lower percentage of black/African-American, Hispanic/Latino, and Asian/Pacific Islanders; had a lower median income despite a lower unemployment rate, in part because of a lower education level; and were equally likely to have health insurance (Table 1). Furthermore, although each of these same differences also existed in 2008, each widened further in 2016 compared with 2008 (online Supplemental eTable 2).

Counties in which President Trump *increased* the Republican percentage of the presidential vote compared with counties in which Secretary Clinton increased the Democratic percentage of presidential vote were older (41.5 vs. 35.1 years); had higher average percent white non-Hispanic/Latino population (81 vs. 42%), but lower average percentage of black/African-Americans (9 vs. 27%), Hispanics/Latinos (7.4 vs. 25%), and Asians/Pacific Islanders (1.1 vs. 7.1%); a lower average median household income ($46,500 vs. $59,500); and a lower percentage of population age 25 years or older with bachelor’s degree or higher (19 vs. 33%; all *p* < 0.001; Table 2). Furthermore, the differences between these counties in all of these metrics widened significantly (*p* < 0.001) in 2015 compared with 2000. Conversely, the mean 2015 unemployment rate (5.6 vs. 6.1%; *p* = 0.250) and mean percentage with health insurance coverage (91 vs. 89%; *p* = 0.939) were similar in counties in which the percentage vote became more Republican or more Democratic. Interestingly, the median net Republican percentage presidential voting gain was slightly lower (12 vs. 16%) in the 165 counties (with less than 4% of the US population) in which the percentage of white non-Hispanic/Latino population rose than in the other 2947 counties in which it declined.

**Table 2:** Comparisons Between Counties with Trump % 2016 Vote > McCain % 2008 Vote Versus Counties with Clinton % 2016 Vote > Obama % 2008 Vote^*^

County characteristics	Counties with Trump % 2016 vote > McCain % 2008 vote (n = 2530)^†^	Counties with Clinton % 2016 vote > Obama % 2008 vote (n = 108)	Significance level
	Mean (SD)	Mean (SD)	p value^‡^
Median age (years)			
Year 2000	37.5 (3.6)	32.7 (2.8)	<0.001
Year 2015	41.5 (4.9)	35.1 (3.1)	<0.001
Change from 2000 to 2015	4.0 (2.5)	2.4 (1.8)	<0.001
% White, not Hispanic or Latino			
Year 2000	84.5 (17.3)	52.8 (20.3)	<0.001
Year 2015	81.0 (17.8)	42.1 (17.6)	<0.001
Change from 2000 to 2015	–3.5 (3.1)	–10.8 (7.6)	<0.001
% Hispanic or Latino			
Year 2000	4.8 (10.0)	19.6 (22.4)	<0.001
Year 2015	7.4 (11.3)	25.0 (24.6)	<0.001
Change from 2000 to 2015	2.6 (2.6)	5.4 (5.0)	<0.001
% Black or African American			
Year 2000	8.5 (14.1)	23.4 (23.1)	<0.001
Year 2015	9.0 (13.7)	26.9 (24.5)	<0.001
Change from 2000 to 2015	0.5 (1.6)	3.5 (6.3)	<0.001
% Asian or Pacific Islander			
Year 2000	0.7 (2.6)	4.6 (5.6)	<0.001
Year 2015	1.1 (2.6)	7.1 (7.5)	<0.001
Change from 2000 to 2015	0.5 (0.6)	2.5 (2.7)	<0.001
% American Indian or Alaska Native			
Year 2000	1.8 (6.5)	0.6 (0.6)	0.975
Year 2015	2.2 (7.0)	0.9 (0.8)	0.948
Change from 2000 to 2015	0.4 (0.8)	0.3 (0.3)	0.954
Median household income (in $1000s)			
Year 2000	36.1 (7.5)	46.3 (17.1)	<0.001
Year 2015	46.5 (9.9)	59.5 (24.0)	<0.001
Change from 2000 to 2015	10.4 (4.9)	13.2 (9.4)	<0.001
% Unemployment			
Year 2000	4.4 (1.6)	4.6 (2.8)	0.070
Year 2015	5.6 (1.8)	6.1 (3.4)	0.250
Change from 2000 to 2015	1.2 (1.2)	1.6 (1.4)	0.990
% of population age 25 years or older with bachelor’s degree or higher			
Year 2000	14.6 (5.2)	27.1 (13.9)	<0.001
Year 2015	18.7 (6.5)	32.7 (16.2)	<0.001
Change from 2000 to 2015	4.1 (2.6)	5.5 (3.7)	<0.001
% with health insurance			
Year 2000	85.5 (4.8)	82.5 (5.9)	0.005
Year 2015	91.0 (3.7)	89.4 (4.2)	0.939
Change from 2000 to 2015	5.4 (3.4)	6.9 (4.6)	<0.001
Age-adjusted death rate per 100,000 persons			
Year 2000	922.1 (143.6)	895.0 (158.7)	<0.001
Year 2015	848.2 (152.4)	734.6 (183.6)	<0.001
Change from 2000 to 2015	–73.9 (127.2)	–160.4 (165.4)	<0.001
Age-adjusted rate of “deaths of despair” per 100,000 persons^§^			
Year 2000	24.1 (27.7)	19.7 (10.2)	0.007
Year 2015	46.0 (31.9)	28.5 (12.5)	<0.001
Change from 2000 to 2015	21.9 (25.4)	8.8 (14.4)	<0.001

SD, standard deviation

*Excluding 398 counties in which both candidates received a smaller percentage vote in 2016 than in 2008 and including 1 county in which both candidates received a higher percentage vote. There were 3113 counties in 2008 but only 3112 counties in 2016 because of the combination of two counties in Virginia (see online Supplemental eTable 1)

†Excluding 77 counties with missing data on one or more of the county characteristics; including only the 2530 counties without missing data on any county characteristics

‡p value based on t test in random intercept models that accounted for clustering of counties within states

§Using the multiply-imputed data on age-adjusted rate of “deaths of despair,” defined as ICD codes listed in online Supplemental eTable 7. The analysis accounts for imputation uncertainty (see online Supplemental Methods)

### Health Correlates of Presidential Voting

**Figure 1:**
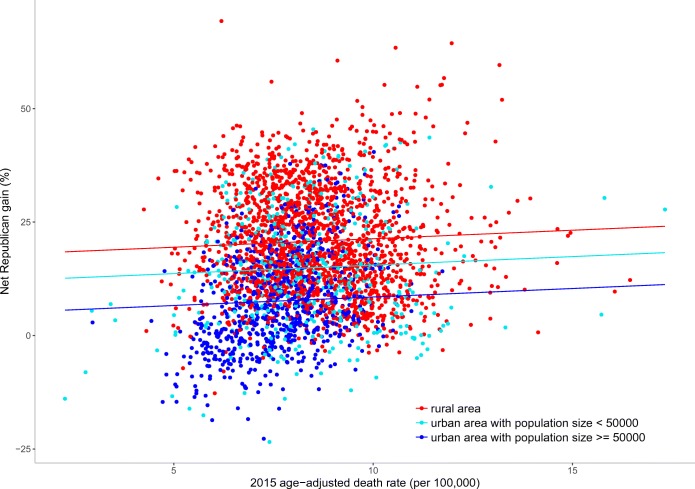
Net Republican gain vs. 2015 age-adjusted death rates. The net Republican gain was greater in rural counties with higher age-adjusted death rates.

**Figure 2:**
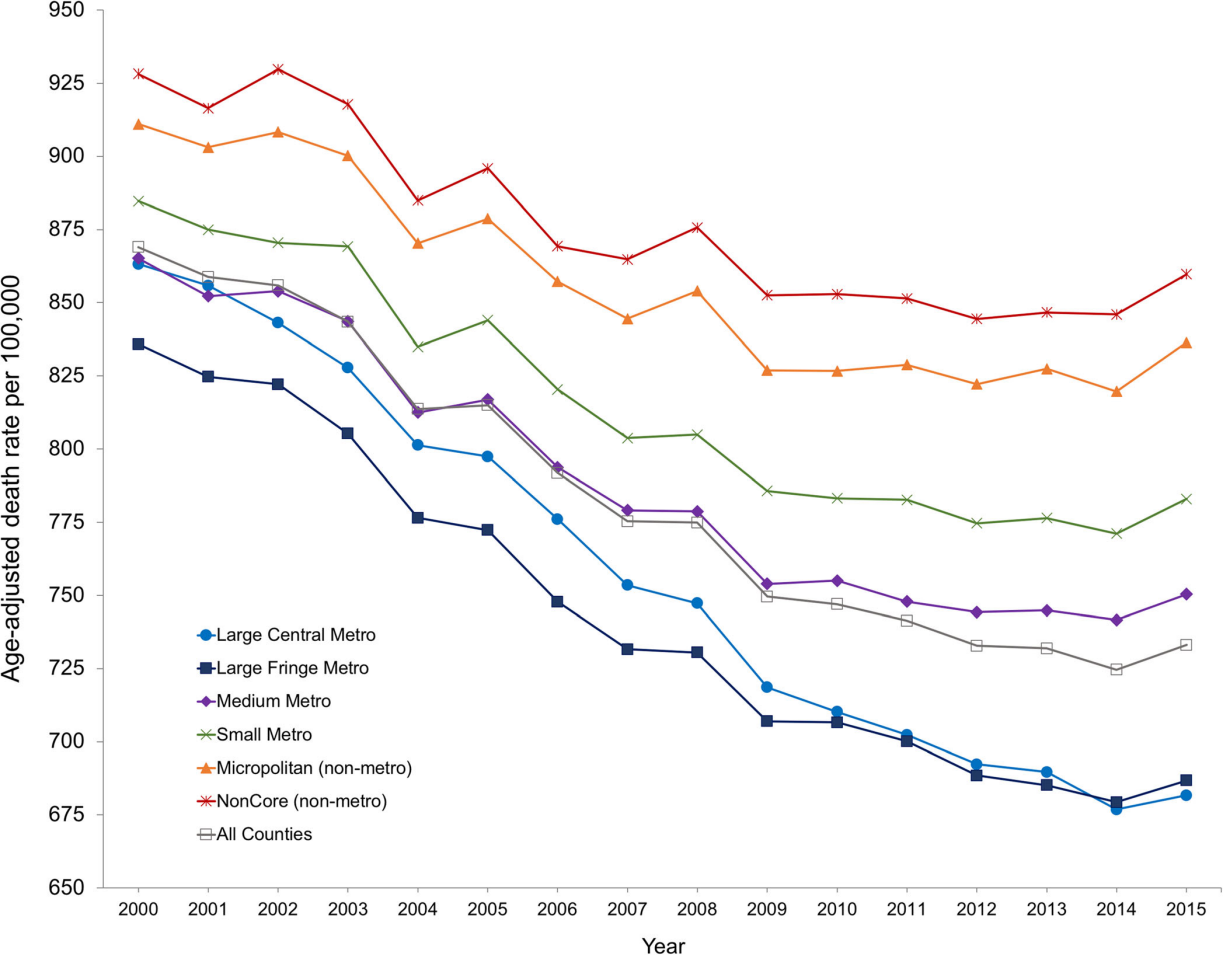
Age-adjusted death rates in counties by urban–rural classification, 2000–2015. Overall age-adjusted death rates are progressively lower in more populous urban counties than in less populous rural counties, and this gap has widened since 2000. Large central metro and large fringe metro counties have a population > 1,000,000; medium metro counties have a population 250,000–999,999; small metro counties have a population < 250,000; micropolitan counties have a non-metro population 10,000–49,999; and noncore non-metro counties have a population < 50,000. Data sources: 2013 NCHS Urban–Rural Classification Scheme for Counties, available at https://www.cdc.gov/nchs/data/series/sr_02/sr02_166.pdf; and the CDC WONDER database, available at https://wonder.cdc.gov/ucd-icd10.html.

The 2015 age-adjusted death rate per 100,000 was significantly higher by 15% (848.2 vs. 734.6) in counties in which President Trump increased the Republican percentage vote over 2008 and by 7.4% (838.8 vs 781.2) in counties that he won. These discrepancies in age-adjusted death rates widened significantly since 2000 because of significantly smaller improvements in counties in which President Trump increased the percentage Republican vote (about 74/100,000; 8%) compared with those in which Senator Clinton increased the percentage Democratic vote (about 160/100,000; 18%). A disproportionate share of the counties with an increased net Republican gain were in the highest quartile of age-adjusted death and had populations below 50,000 (Fig. 1 and online Supplemental eFig. 2). This trend was a function of the growing discrepancies in age-adjusted death rates in more populous counties, in which Secretary Clinton increased the Democratic percentage vote, vs. less populous counties in which President Trump increased the Republican percentage vote (Fig. 2). Age-adjusted death rates due to alcohol, drugs, and suicide increased nationally between 2000 and 2015, but they increased 2.5 times more in counties with an increasing Republican percentage vote (24.1/100,000 to 46.0/100,000) than in counties with an increased Democratic percentage vote (19.7/100,000 to 28.5/100,000). This greater increase in deaths of despair accounted for 15% of the 2015 difference in death rates between these two groups of counties.

### Multivariable Analyses

A county’s net Republican percentage gain in presidential voting in 2016 vs. 2008 was independently correlated with a smaller decrease in age-adjusted death rates even after considering its very significant relationship with a county’s smaller population; less urban status; less increase in its percentage of black/African-American, Hispanic/Latino, and Asian/Pacific Islander population; and less increase in its median income, its unemployment rate, its education level, and its health insurance rate since 2000 (Table 3). Age-adjusted rates of deaths of despair were not significantly related to net Republican percentage gain after adjusting for age-adjusted death rates from other causes and these other covariates (online Supplemental eTable 3).

**Table 3:** Multivariable Random-Effects Model Examining the Impacts of the Changes in County-Level Demographic, Socioeconomic, and Health Characteristics between 2015 and 2000 on the Net Republican Gain between the 2016 and 2008 Elections^*^

Fixed Effects	Magnitude correlating with each 1% net Republican gain	p value^†^	More Republican gain in county if:
Change in median age	44.51 years	0.753	More increase
Change in % Black or African American	–3.86%	<0.001	Less increase
Change in % Hispanic or Latino	–2.35%	<0.001	Less increase
Change in % Asian or Pacific Islander	–0.72%	<0.001	Less increase
Change in median income	–$7,504	<0.001	Less increase
Change in unemployment rate	–3.49%	0.047	Less increase
Change in % of population age 25 years or older with bachelor’s degree or higher	–1.99%	<0.001	Less increase
Change in health insurance rate	–2.56%	<0.001	Less increase
Change in age-adjusted death rate	131/100,000 persons	<0.001	Less decrease
Rural vs. urban area	Given all the other covariates, counties in rural areas have on average 3.53% higher net Republican gain than counties in urban areas	<0.001	More rural
Effect of log10(county population) for counties with size ≤ 50,000	Given all other covariates, every 10-fold increase in population size will lead to 2.97% lower net Republican gain for counties with population ≤ 50,000	<0.001	Lower population
Effect of log10(county population) for counties with size > 50,000	Given all other covariates, every 10-fold increase in population size will lead to 7.21% lower net Republican gain for counties with population > 50,000	<0.001	Lower population
**Random Effects**			
Variance of states in the random intercept			61.87
Variance of states in the random slope associated with change in age-adjusted death rate			0.70
Correlation between the random intercept and random slope			0.49
R^2^ with variance explained by both fixed and random effects			0.68
R^2^ with variance explained by fixed effects only			0.31

*Excluding 88 counties with missing data on one or more of the county characteristics

†*p* value based on t test in the random effects model that accounted for clustering of counties within states

### Electoral Implications

The average independent nationwide impact of age-adjusted death rates was that a 131/100,000 improvement would result in a 1% lower net Republican percentage vote gain in 2016 compared with 2008 in our primary analysis, 73/100,000 in the weighted random effects model (online Supplemental eTable 4), and 124/100,000 in the random effects principal component analysis (online Supplemental eTable 5), and 76/100,000 in the random effects model with net Republican percentage gain between the 2000 and 2016 elections (online Supplemental eTable 6).

In the three key swing states with the smallest victory margins for President Trump (Michigan, 0.22%; Pennsylvania, 0.72%; and Wisconsin, 0.76%), the differences in death rates associated with a 1% net Republican percentage gain were smaller than average—80/100,000 in Michigan, 61/100,000 in Pennsylvania, and 88/100,000 in Wisconsin in our primary analysis. Although correlation does not infer causality, these three states and the election could theoretically have shifted to Secretary Clinton if their 2015 age-adjusted death rates had been 18, 44, and 67 lower per 100,000, respectively, in our more conservative, primary analysis.

## DISCUSSION

American presidential elections are likely influenced by economic^25^ and social trends,^26^ which can drive swing voters from one party to another^3^ and might increase voter turnout. Not surprisingly, we found that counties with no or slower gains in income were significantly more likely to vote for President Trump in 2016 than Senator McCain in 2008. President Trump’s emphasis on non-Hispanic white voters was reflected by his better performance in counties with smaller increases in ethnic and racial diversity.

Mortality rates among lower-income, rural, non-Hispanic white Americans have been rising, even as they are declining in blacks and Hispanics.^27^ By the 2016 election, counties won by President Trump had a 7.4% higher age-adjusted death rate than counties won by Secretary Clinton; however, counties in which President Trump’s percentage vote in 2016 was higher than Senator McCain’s percentage vote in 2008 had a 15% higher age-adjusted death rate. Age-adjusted death rates remained a significant correlate of a county’s net Republican percentage vote gain between 2008 and 2016 even when adjusting for the county population, rural-urban status, median family income, educational levels, percentage of population that is non-white, and rates of unemployment and health insurance coverage.

Our findings are generally consistent with recent analyses by Bor^8^ and by Wasfy and colleagues^7^ but expand them in important ways. Wasfy et al. reported that voting changes between the 2012 and 2016 US presidential elections were strongly and independently correlated with 2014 county-level health (as measured by a composite of age-adjusted death rate, teen birth rate, violent crime rate, primary care physician/100,000 people, and self-reported survey data on average health care costs; physically unhealthy or mentally unhealthy days in the past 30 days; percent overweight or obese; percent diabetic; and percent with food insecurity) after adjusting for essentially the same socioeconomic and demographic covariates we used.^7^ Rather than focus on static health predictors, we focused on changes over a 15-year time span. Bor reported that the magnitude of improvement in life expectancy between 1980 and 2014 was inversely correlated with a county’s voting share for President Trump in 2016 compared with Senator McCain in 2008 but that this relationship became non-significant after adjusting for state-wide effects as well as a county’s 2014 urban-rural status, economic measures, educational level, and racial/ethnic composition. He concluded that these geographic and socioeconomic measures are driving changes in both voting patterns and life expectancy. However, he considered these state-level measures at one point in time, not their changes over time. Bilal et al. noted that counties with increasing all-cause mortality in persons age 45–54 years were more likely to vote Democratic in 2008 and 2012 but Republican in 2016, with each 15.2/100,000 increase associated with a 1% swing.^9^ Our analysis therefore adds important nuance to these excellent prior reports. We show that changes over time in the age-adjusted death rates correlate independently with changes in county-level voting after adjusting for changes in other socioeconomic and demographic measures.

By focusing on county-level changes, we eliminated several potential biases. For example, President Trump targeted populations at higher risk of mortality, but we showed that net declines in important socioeconomic indicators, and not just the levels of the indicators themselves, correlate with mortality and voting behavior. Counties with lower baseline income or higher baseline mortality would likely continue to rank low on these measures over time, and controlling for these factors, as Bor did, could cause their effect to disappear. Our data emphasize the importance of relative improvement or decline in county-level factors on voting behavior. Our emphasis on changes in voting, in death rates, and in all other predictive variables is consistent with a substantial literature showing that a person’s happiness is less dependent on their current status than on recent changes in their status—whether economic, social, or health-related—because people tend to adapt, at least in part, to their new status over time by recalibrating their aspirations and expectations.^28, 29^

In the USA, life expectancy declined from 78.9 years in 2012 to 78.6 years in 2016. Declines among younger, lower-income, non-Hispanic whites, especially those without a bachelor’s degree, more than offset gains among African-Americans.^6, 10, 12, 30, 31^ Increases in deaths of despair, which approximately doubled between 2000 and 2015, contributed to this decline in life expectancy.^11, 32–34^ However, a variety of socioeconomic, ethnic, behavioral, and metabolic factors are important,^35^ as are wealth^36, 37^ and education.^38^ Although death rates are not the only marker of health and well-being, they might be a marker of relative despair. If so, it is not surprising that deaths related to alcohol, drugs, and suicides rose by 2.5 times as much in counties with a Republican percentage gain compared with counties with a Democratic percentage gain since 2000.

Health insurance coverage, which might save lives^39^ but cannot fully offset the effects of lifestyle and poverty, increased in counties that became more Democratic and that had greater reductions in death rates.^40^ Conversely, counties with higher death rates voted for a Republican presidential candidate whose party promised to repeal the Affordable Care Act. These counter-intuitive findings have been broadly discussed in the sociological literature and may be due to political messaging.

Our analyses have several limitations. First, county-level correlations do not guarantee that individuals with these characteristics changed their votes or preferentially voted in one election or another. Second, available data sources cannot prove a causal chain of events, and voting correlations do not equate with causality.

Rates of “deaths of despair,” which are related to alcohol, drugs, and suicide, are increasing at a time when the sum of all other causes of death are declining. Although President Trump over-performed in counties with higher rates of deaths of despair,^41^ such deaths represent less than 5% of age-adjusted death rates and, at least in our analyses, do not, in and of themselves, explain a substantial proportion of the voting change. It is possible, however, that a more expansive consideration of all deaths related to drugs, alcohol, depression, and anxiety might find a stronger independent relationship. Unfortunately, the notorious unreliability of death certificates^42^ makes such estimates problematic.

Our primary analysis, as well as three alternative sensitivity analyses, found that relatively modest incremental reductions in age-adjusted, county-level death rates could hypothetically have swung Michigan, Pennsylvania, and Wisconsin and, hence, the election to Secretary Clinton. Even in our most conservative, primary analysis, these age-adjusted death rate reductions were plausible and potentially obtainable—18/100,000 lower in Michigan (to a rate between Pennsylvania and Montana), 44/100,000 lower in Pennsylvania (to a rate between Oregon and Iowa), and 67/100,000 lower in Wisconsin (to a rate between Minnesota and New York). These findings are consistent with a recent county-level analysis suggesting that Secretary Clinton hypothetically could have carried Michigan if its prevalence of diabetes was 7% lower, Pennsylvania if an additional 8% of its residents engaged in regular physical exercise, and Wisconsin if its rate of heavy drinking was 5% lower.^5^ Death rates may be important markers of the dissatisfaction, discouragement, hopelessness, and fear of cultural displacement that contributed to President Trump’s appeal, especially to the non-urban, white working class.^43^

## Electronic supplementary material

ESM 1 (DOCX 527 kb)
