# Supplementary material for: Independent Relationship of Changes in Death Rates with Changes in US Presidential Voting
Source: J Gen Intern Med. 2018 Sep 5;34(3):363–71. doi: 10.1007/s11606-018-4568-6 (PMC6420486; doi:10.1007/s11606-018-4568-6)
Supplement: Supplementary file 1 — (DOCX 527 kb) [file 11606_2018_4568_MOESM1_ESM.docx]

**Independent Relationship of Changes in Death Rates with Changes in US Presidential Voting**

**Goldman L, Lim MP, Chen Q, Jin P, Muennig P, Vagelos A**

***Journal of General Internal Medicine***

**2018**

**SUPPLEMENTARY APPENDICES**

**Supplemental eFigure 1:** The Number of Counties with a Net Percentage Republican Gain or a Net Percentage Democratic Gain, 2016 vs. 2008

**Supplemental eFigure 2:** Age-Adjusted Death Rates in the Context of the Correlation of County Size with Net Republican vs. Net Democratic Gain in Percentage of Presidential Vote, 2008­–2016

**Supplemental eTable 1:** Reconciliation of the Reported 3141 Total Counties in the US vs. the Number of Counties Reported in Table 1 (3113 in 2008 and 3112 in 2016)

**Supplemental eTable 2:** Comparisons Between Counties Won by the Republican or Democratic Presidential Candidate in Election Years 2008 and 2016

**Supplemental eTable 3:** The Multivariable Random-Effects Model in Table 3, but Now Including Both the Age-adjusted Rate of “Deaths of Despair” and the Age-Adjusted Rate of “All Other” Deaths

**Supplemental eTable 4:** Multivariable Random-Effects Model in Table 3, but Now Weighted by a County’s Population Size

**Supplemental eTable 5:** Multivariable Random-Effects Model in Table 3, but Now Using Principal Component Analysis on the Changes in Age, Race, Income, Unemployment, Education, and Health Insurance from 2000 to 2015

**Supplemental eTable 6:** Multivariable Random-Effects Model in Table 3, but Now Using Net Republican Gain from 2000 to 2016 as the Outcome Variable

**Supplemental eTable 7:**  International Statistical Classification of Diseases and Related Health Problems, Tenth Revision (ICD-10) Codes Classified as Deaths of Despair^*^

**Supplemental Methods:** Multiple Imputation Method for Imputing Missing Age-adjusted Rates of “Deaths of Despair” for Counties with Suppressed Death Counts


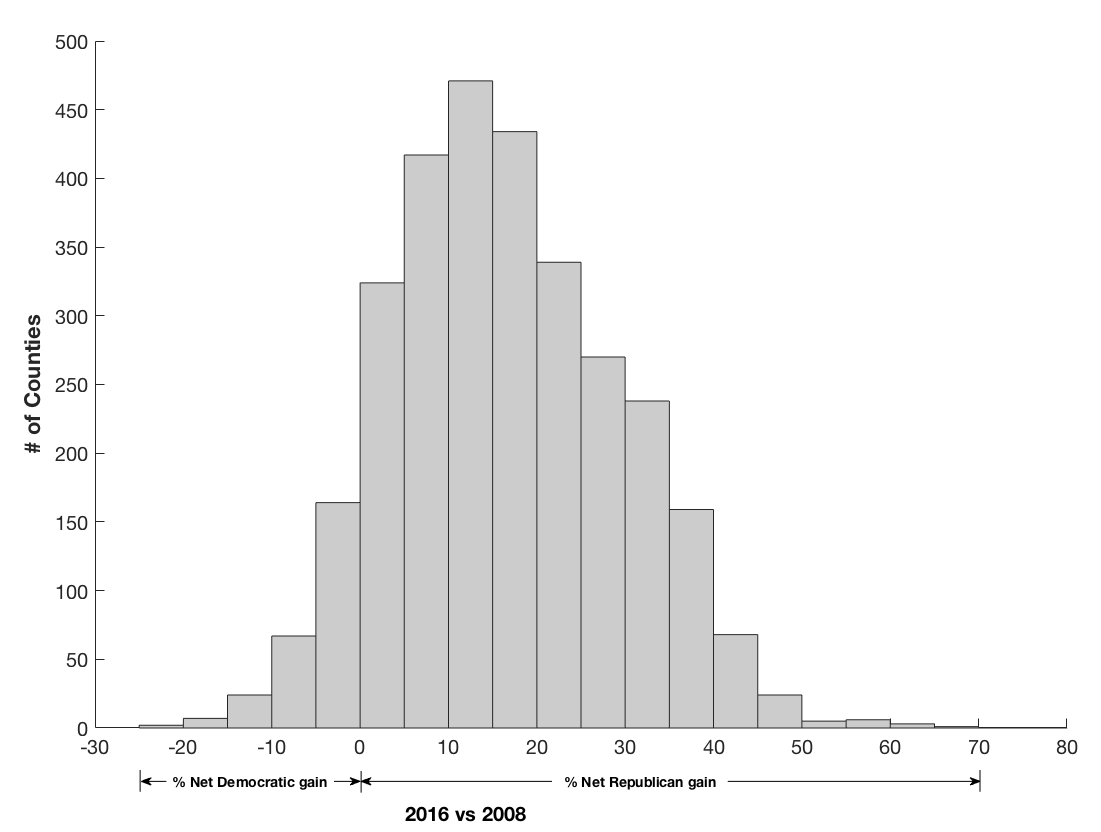


**Supplemental eFigure 1. The Number of Counties with a Net Percentage Republican Gain or a Net Percentage Democratic Gain, 2016 vs. 2008.**

398 counties had declines in percentage of votes for both parties because of increased votes to other party candidates, and 1 county had an increase in percentage to both parties.

**Supplemental eFigure 2.** **Age-Adjusted Death Rates in the Context of the Correlation of County Size with Net Republican vs. Net Democratic Gain in Percentage of Presidential Vote, 2008–2016.**

**Supplemental eTable 1 Reconciliation of the Reported 3141 Total Counties in the US vs. the Number of Counties Reported in Table 1 (3113 in 2008 and 3112 in 2016)**

| **Categories of counties and county-equivalents in the US^*^** | **Included in county voting data?** | **2008** | **2016** |
| --- | --- | --- | --- |
| 3007 entities named “County” | Yes | 3007 | 3007 |
| 16 Boroughs in Alaska | No^†^ | -- | -- |
| 11 Census Areas in Alaska (for areas not organized into Boroughs) | No^†^ | -- | -- |
| 64 Parishes in Louisiana | Yes | 64 | 64 |
| 42 Independent cities |  |  |  |
| - 1 in Maryland (Baltimore) | Yes | 1 | 1 |
| - 1 in Missouri (St. Louis) | Yes | 1 | 1 |
| - 1 in Nevada (Carson City) | Yes | 1 | 1 |
| - remainder in Virginia | Yes^‡^ | 39 | 38 |
| 1 District – the Federal District or District of Columbia | Yes | 1 | 1 |
| **Total 3141** |  | **3113^§^** | **3112^§^** |

*^*^ Source: U.S. Geological Survey.* [*https://www.usgs.gov/faqs/how-many-counties-are-there-united-states*](https://www.usgs.gov/faqs/how-many-counties-are-there-united-states)*. Accessed June 5, 2018.*

*^†^ Election data are not available at the county level because Alaska’s voting regions comprise 40 districts rather than its 27 county-equivalents (boroughs and census areas)*

*^‡^ The independent city of Bedford, VA, (FIPS code 51515) was designated a town in 2013 and added to Bedford County (FIPS code 51019)*

*^§^ Voting data for Kalawao County, HI, (FIPS code 15005; population ~90) are not reported*

**Supplemental eTable 2 Comparisons Between Counties Won by the Republican or Democratic Presidential Candidate in Election Years 2008 and 2016**

|  | **2016 year election** | | | **2008 year election** | | | **2016 vs. 2008** | |  |
| --- | --- | --- | --- | --- | --- | --- | --- | --- | --- |
| **County characteristics in 2015 and 2007^*^** | **Counties Won by President Trump (n=2541)** | **Counties Won by Secretary Clinton (n=482)** | **Significance level** | **Counties Won by Senator McCain (n=2159)** | **Counties Won by President Obama (n=864)** | **Significance level** | | **Significance level of difference in 2016 vs. 2008** | |
|  | Mean (SD) | Mean (SD) | *p* value^†^ | Mean (SD) | Mean (SD) | *p* value^†^ | | *p* value^‡^ | |
| Median age (years) | 41.2 (5.0) | 37.6 (5.4) | < 0.001 | 38.7 (4.3) | 37.5 (4.6) | < 0.001 | | < 0.001 | |
| White, not Hispanic or Latino (%) | 82.2 (15.3) | 55.5 (24.9) | < 0.001 | 83.7 (14.7) | 70.9 (25.5) | < 0.001 | | < 0.001 | |
| Black or African American (%) | 7.6 (10.5) | 23.0 (23.9) | < 0.001 | 7.5 (10.7) | 14.9 (21.0) | < 0.001 | | < 0.001 | |
| Hispanic or Latino (%) | 7.9 (11.1) | 15.5 (21.1) | < 0.001 | 6.9 (10.5) | 9.8 (17.0) | < 0.001 | | 0.024 | |
| Asian or Pacific Islander (%) | 1.1 (1.2) | 4.6 (7.3) | < 0.001 | 0.8 (1.1) | 2.5 (5.3) | < 0.001 | | < 0.001 | |
| Median income (in $1000s) | 47.8 (10.7) | 52.0 (18.3) | < 0.001 | 41.7 (9.7) | 45.3 (13.3) | 0.744 | | < 0.001 | |
| Unemployment rate (%) | 5.4 (1.8) | 6.2 (2.4) | < 0.001 | 4.7 (1.5) | 5.3 (1.9) | < 0.001 | | 0.001 | |
| Population with bachelor's degree or higher age 25 years or older (%) | 19.2 (7.1) | 29.7 (13.5) | < 0.001 | 16.9 (6.5) | 23.0 (11.0) | < 0.001 | | < 0.001 | |
| Health insurance rate (%) | 90.9 (3.7) | 91.5 (3.8) | 0.771 | 84.6 (4.6) | 86.7 (4.5) | 0.012 | | 0.129 | |
| Age-adjusted death rate (per 100,000) | 838.8 (148.2) | 781.2 (190.1) | < 0.001 | 854.4 (142.0) | 810.4 (162.8) | 0.046 | | < 0.001 | |

*SD, standard deviation*

*^*^ Excludes 89 counties with missing data on one or more county characteristics*

*^†^ p value based on t test in the random intercept models that accounted for clustering of counties within states*

*^‡^ p value based on t test in the 3-level random intercept model that accounted for clustering of counties within states and correlation of two years of measurements for each county*

**Supplemental eTable 3 The Multivariable Random-Effects Model in Table 3, but Now Including Both the Age-adjusted Rate of “Deaths of Despair” and the Age-Adjusted Rate of “All Other” Deaths**

| **Fixed Effects:** | **Magnitude correlating with each 1% net Republican gain** | ***p*** **value^*^** | **More Republican gain in county if:** |
| --- | --- | --- | --- |
| **Change in median age** | 40.19 years | 0.729 | More increase |
| **Change in % Black or African American** | – 3.97% | < 0.001 | Less increase |
| **Change in % Hispanic or Latino** | – 2.38% | < 0.001 | Less increase |
| **Change in % Asian or Pacific Islander** | – 0.72% | < 0.001 | Less increase |
| **Change in median income** | – $7,341 | < 0.001 | Less increase |
| **Change in unemployment rate** | – 3.46% | 0.045 | Less increase |
| **Change in % of population age 25 years or older with bachelor’s degree or higher** | – 1.99% | < 0.001 | Less increase |
| **Change in health insurance rate** | – 2.60% | < 0.001 | Less increase |
| **Change in age-adjusted rate of “deaths of despair”** | 99/100,000 persons | 0.357 | More increase |
| **Change in age-adjusted rate of “all other deaths”** | 131/100,000 persons | < 0.001 | Less decrease |
| **Rural vs. urban area** | Given all the other covariates, counties in rural areas have on average 3.57% higher net Republican gain than counties in urban areas | < 0.001 | More rural |
| **Effect of log10(county population) for counties with size ≤ 50,000** | Given all other covariates, every 10-fold increase in population size will lead to 2.97% lower net Republican gain for counties with population ≤ 50,000 | < 0.001 | Lower population |
| **Effect of log10(county population) for counties with size > 50,000** | Given all other covariates, every 10-fold increase in population size will lead to 7.17% lower net Republican gain for counties with population > 50,000 | < 0.001 | Lower population |

***^*^*** *p value based on t test in the random effects model that accounted for clustering of counties within states*

**Supplemental eTable 4 Multivariable Random-Effects Model in Table 3, but Now Weighted by a County’s Population Size**

| **Fixed Effects:** | **Magnitude correlating with each 1% net Republican gain** | ***p* value^*^** | **More Republican gain in county if:** |
| --- | --- | --- | --- |
| **Change in median age** | 3.70 years | < 0.001 | More increase |
| **Change in % Black or African American** | – 3.27% | < 0.001 | Less increase |
| **Change in % Hispanic or Latino** | – 2.03% | < 0.001 | Less increase |
| **Change in % Asian or Pacific Islander** | – 0.48% | < 0.001 | Less increase |
| **Change in median income** | $23,615 | 0.226 | More increase |
| **Change in unemployment rate** | – 3.24% | 0.060 | Less increase |
| **Change in % of population age 25 years or older with bachelor’s degree or higher** | – 1.31% | < 0.001 | Less increase |
| **Change in health insurance rate** | – 2.79% | < 0.001 | Less increase |
| **Change in age-adjusted death rate** | 73/100,000 persons | < 0.001 | Less decrease |
| **Rural vs. urban area** | Given all the other covariates, counties in rural areas have on average 7.01% higher net Republican gain than counties in urban areas | < 0.001 | More rural |

***^*^*** *p value based on t test in the random effects model that accounted for clustering of counties within states*

**Supplemental eTable 5 Multivariable Random-Effects Model in Table 3, but Now Using Principal Component Analysis on the Changes in Age, Race, Income, Unemployment, Education, and Health Insurance from 2000 to 2015**

| **Fixed Effects^*^:** | **Magnitude correlating with each 1% net Republican gain** | ***p* value^†^** | **More Republican gain in county if:** |
| --- | --- | --- | --- |
| **Change in age-adjusted death rate** | 124/100,000 persons | < 0.001 | Less decrease |
| **Rural vs. urban area** | Given all the other covariates, counties in rural areas have on average 3.59% higher net Republican gain than counties in urban areas | < 0.001 | More rural |
| **Effect of log10(county population) for counties with size ≤ 50,000** | Given all other covariates, every 10-fold increase in population size will lead to 2.66% lower net Republican gain for counties with population ≤ 50,000 | < 0.001 | Lower population |
| **Effect of log10(county population) for counties with size > 50,000** | Given all other covariates, every 10-fold increase in population size will lead to 7.06% lower net Republican gain for counties with population > 50,000 | < 0.001 | Lower population |

***^*^*** *Model adjusted for the first six principal components, which accounted for around 85% variation of the change in age, race, income, unemployment, education, and health insurance data*

***^†^*** *p value based on t test in the random effects model that accounted for clustering of counties within states*

**Supplemental eTable 6 Multivariable Random-Effects Model in Table 3, but Now Using Net Republican Gain from 2000 to 2016 as the Outcome Variable**

| **Fixed Effects:** | **Magnitude correlating with each 1% net Republican gain** | ***p* value^*^** | **More Republican gain in county if:** |
| --- | --- | --- | --- |
| **Change in median age** | – 1.73 years | < 0.001 | Less increase |
| **Change in % Black or African American** | – 0.85% | < 0.001 | Less increase |
| **Change in % Hispanic or Latino** | – 1.29% | < 0.001 | Less increase |
| **Change in % Asian or Pacific Islander** | – 0.43% | < 0.001 | Less increase |
| **Change in median income** | $20,392 | 0.426 | More increase |
| **Change in unemployment rate** | 3.11% | 0.211 | More increase |
| **Change in % of population age 25 years or older with bachelor’s degree or higher** | – 1.25% | < 0.001 | Less increase |
| **Change in health insurance rate** | – 1.23% | < 0.001 | Less increase |
| **Change in age-adjusted death rate** | 76/100,000 persons | < 0.001 | Less decrease |
| **Rural vs. urban area** | Given all the other covariates, counties in rural areas have on average 6.39% higher net Republican gain than counties in urban areas | < 0.001 | More rural |
| **Effect of log10(county population) for counties with size ≤ 50,000** | Given all other covariates, every 10-fold increase in population size will lead to 3.47% lower net Republican gain for counties with population ≤ 50,000 | < 0.001 | Lower population |
| **Effect of log10(county population) for counties with size > 50,000** | Given all other covariates, every 10-fold increase in population size will lead to 11.10% lower net Republican gain for counties with population > 50,000 | < 0.001 | Lower population |
| ***^*^*** *p value based on t test in the random effects model that accounted for clustering of counties within states* | | | |

**Supplemental eTable 7 International Statistical Classification of Diseases and Related Health Problems, Tenth Revision (ICD-10) Codes Classified as Deaths of Despair^*^**

| D52.1, D59.0, D59.2, D61.1, D64.2  E06.4, E16.0, E23.1, E24.2, E24.4, E27.3, E66.1  F10-F19  G21.1, G24.0, G25.1, G25.4, G25.6, G31.2, G44.4, G62.0, G62.1, G72.0, G72.1  I42.6, I95.2  J70.2, J70.3, J70.4  K29.2, K70, K85.2, K85.3, K86.0  L10.5, L27.0, L27.1  M10.2, M32.0, M80.4, M81.4, M83.5, M87.1  R50.2  R78.0-R78.5  U03.0, U03.9  X40-X45  X60-X84  Y10-Y15  Y87.0 |
| --- |
| *ICD, International Statistical Classification of Diseases and Health Related Problems*  *^*^ ICD-10 codes taken from the Centers for Disease Control and Prevention’s categories for Drug/Alcohol Induced Causes, and for the Injury Intent category of suicide; overlapping codes across these categories were removed (i.e. X60-65 suicide deaths due to drugs/alcohol). Data methodology from Trust for America’s Health. Pain in the nation update: deaths from alcohol, drugs and suicide reach the highest level ever recorded. February 2018.* [*http://healthyamericans.org/assets/files/TFAH-2018-PainNationUpdateBrief-FINAL.pdf*](http://healthyamericans.org/assets/files/TFAH-2018-PainNationUpdateBrief-FINAL.pdf)*. Accessed June 5, 2018.* |

**Supplemental Methods: Multiple Imputation Method for Imputing Missing Age-adjusted Rates of “Deaths of Despair” for Counties with Suppressed Death Counts**

We imputed the age-adjusted rates of “deaths of despair” in 2000 and 2015 for counties with missing data on this variable. For counties with missing age-adjusted rates of “deaths of despair” but observed death counts for alcohol, drugs, or suicide, we imputed the age-adjusted death rates by the crude death rates without age adjustment, calculated using death counts divided by county population sizes. For counties with suppressed death counts, in which a death count is suppressed if the count is smaller than 10, we imputed the missing age-adjusted rates of “deaths of despair” using a Bayesian imputation model. We built a multilevel left-censored Bayesian imputation model, where the two years of age-adjusted rates of “deaths of despair” are nested under each county, and counties are nested under states, so the imputation model allowed the age-adjusted rates of “deaths of despair” to vary by counties and states. Moreover, a left-censored regression allowed us to incorporate data from both counties with observed age-adjusted rates of “deaths of despair” and counties with suppressed death counts, in which the age-adjusted rates of “deaths of despair” are left-censored at 9 divided by the county population size. Since the observed age-adjusted overall death rates follows a normal distribution, we assumed that the age-adjusted rate of “deaths of despair” also followed a normal distribution. The imputation model used county-level measures as covariates, including a county’s urban/rural status, population size, median age, race, income, unemployment rate, education, health insurance, and age-adjusted overall death rate, as well as election results in the two years. The Bayesian imputation model was implemented using RStan (Stan Development Team) by assuming uniform priors for regression coefficients and weakly informative half-Cauchy priors for variance parameters. The convergence of the Markov chain Monte Carlo simulation was checked using the convergence measure R-hat that suggests the chains mix well if close to 1. We generated 50 imputations for each missing age-adjusted rate of “deaths of despair” and created 50 imputed datasets. We conducted statistical analyses in each of the 50 imputed datasets. The analysis results from the multiple imputations were then combined using Rubin’s multiple imputation combining rules, which account for both within- and between-imputation uncertainties (Rubin DB. Multiple Imputation for Nonresponse in Surveys. Wiley: New York; 1987). The analyses were implemented using the “pool” function in the “mice” package in R.
